# Supplementary material for: Content-rich biological network constructed by mining PubMed abstracts
Source: BMC Bioinformatics. 2004 Oct 8;5:147. doi: 10.1186/1471-2105-5-147 (PMC528731; doi:10.1186/1471-2105-5-147)
Supplement: Additional File 2 — The original results of the above study (non-essential files are deleted to keep the file size under the limit set by BMC bioinformatics). [file 1471-2105-5-147-S2.bz2 › chilibotAdditionalFile2/dip10/281ID2498083E1104/html/FOS_JUNB.html]

 


 **FOS** and **JUNB** 
  
Found 830 abstracts in PubMed, retrieved 10.  
 

 What does Google say? 
 PDF only 
| .edu only 

---

**Interactive relationship** (e.g. stimulation, inhibition, etc)

**Non-interactive relationship** (e.g. studied together, co-existance, homology, etc.)

- In injured animals without SD, only a short lasting response of c  **fos** ,  **junB** , c jun and MKP 1 mRNAs as well as c  **Fos**  protein was bilaterally found in the piri form cortex, and with ipsilateral dominance the dentate gyrus and hippocampal CA3 4 fields at 1 h after lesioning.  Ref: 12671253 Restor Neurol Neurosci< MedlineTA>Restor Neurol Neurosci,
- Supershift electrophoretic mobility shift assay indicated that  **JunB** , JunD, and c  **Fos**  components of AP 1 were particularly affected.  Ref: 12857751 J Biol ChemJ Biol Chem,
- Levels were highest shortly after transient DC shifts, and decreased thereafter mono exponentially with half lives of 4 5 and 58 min for c  **fos** ,  **junB**  and MKP 1 mRNAs, respectively.  Ref: 12671253 Restor Neurol Neurosci< MedlineTA>Restor Neurol Neurosci,
- Acute injection of morphine induces expression of the immediate early genes c  **Fos**  and  **JunB**  in several forebrain regions of the rat, in part through an N methyl D aspartate NMDA receptor dependent mechanism.  Ref: 12809699 Neuroscience< MedlineTA>Neuroscience,
- In a Gal4 fusion assay, Pdcd4 specifically inhibited activation of c Jun and c  **Fos**  activation domains, but did not inhibit activation of  **JunB** , JunD, Fra 1, or Fra 2.  Ref: 12802278 Oncogene, 2003
- Using specific polyclonal antisera against c  **Fos** ,  **JunB** , c Jun and JunD, we tried to identify the candidate transcription factors of the immediate early gene family which may contribute to the molecular processes during contextual memory reconsolidation.  Ref: 12882314 Genes Brain Behav, 2003
- In contrast, mice exposed and re exposed only to the context, without foot shock, did not show behavioral signs of contextual fear conditioning and exhibited significantly less expression of c  **Fos**  and  **JunB**  in CA1 neurons.  Ref: 12882314 Genes Brain Behav, 2003
- In these mice context dependent memory retrieval evoked a marked induction of c  **Fos**  and  **JunB** , but not of c Jun and JunD, in pyramidal CA1 neurons of the dorsal hippocampus.  Ref: 12882314 Genes Brain Behav, 2003
- Genes that were commonly down regulated in both PB T cells and Jurkat T cells included CDK inhibitors p16, p19, p27, proapoptotic caspases, and the transcription factors c  **fos**  and jun B  [ **JUNB** ] .  Ref: 12808112 Lab Invest, 2003
- Towards this end, we characterized the effect pharmacological disruption of the MAPK cascade has on the expression of the immediate early genes c  **Fos** ,  **JunB**  and EGR 1.  Ref: 12752379 Eur J Neurosci, 2003
- Mechanistically, sodium valproate and pyridoxine significantly attenuated domoic acid induced increase in levels of glutamate, increase in levels of calcium influx, decrease in levels of gamma aminobutyric acid and increase in levels of the protooncogenes c  **fos** , jun B  [ **JUNB** ]  and jun D.  Ref: 12686137 Biochim Biophys Acta, 2003
- Memory retrieval after contextual fear conditioning induces c  **Fos**  and  **JunB**  expression in CA1 hippocampus.  Ref: 12882314 Genes Brain Behav, 2003
- The expression of c  **fos** ,  **junB**  and MKP 1 mRNAs was closely related to the time interval between the last spreading depression and the end of the experiments.  Ref: 12671253 Restor Neurol Neurosci< MedlineTA>Restor Neurol Neurosci, 2003
